# Using Progress Feedback to Enhance Treatment Outcomes: A Narrative Review

**DOI:** 10.1007/s10488-024-01381-3

**Published:** 2024-05-11

**Authors:** Kim de Jong, Susan Douglas, Miranda Wolpert, Jaime Delgadillo, Benjamin Aas, Bram Bovendeerd, Ingrid Carlier, Angelo Compare, Julian Edbrooke-Childs, Pauline Janse, Wolfgang Lutz, Christian Moltu, Samuel Nordberg, Stig Poulsen, Julian A. Rubel, Günter Schiepek, Viola N. L. S. Schilling, Maartje van Sonsbeek, Michael Barkham

**Affiliations:** 1 Clinical Psychology Unit, Institute of Psychology, Leiden University, Wassenaarseweg 52, Leiden, 2333 AK The Netherlands; 2 Department of Leadership, Policy and Organizations, Vanderbilt University, Nashville, TN USA; 3 Division of Psychology and Language Sciences, Department of Clinical, Education and Health Psychology, University College London, United Kingdom, UK; 4 Clinical and Applied Psychology Unit, Department of Psychology, University of Sheffield, Sheffield, UK; 5 Department of Child and Adolescent Psychiatry, Psychosomatics and Psychotherapy, University Hospital, LMU Munich, Munich, Germany; 6 Faculty of Psychology and Educational Sciences, LMU Munich, Munich, Germany; 7 Department of Clinical Psychology and Experimental Psychopathology, Faculty of Behavioural and Social Sciences, University of Groningen, Groningen, The Netherlands; 8 Dimence, Center for mental health care, Deventer, The Netherlands; 9 Department of Psychiatry, Leiden University Medical Centre, Leiden, The Netherlands; 10 Department of Human and Social Sciences, University of Bergamo, Bergamo, Italy; 11 Evidence Based Practice Unit, Anna Freud National Centre for Children and Families, University College London, London, UK; 12 Pro Persona Research, Renkum, The Netherlands; 13 Department of Psychology, University of Trier, Trier, Germany; 14 District General Hospital of Førde, Førde, Norway; 15 Department of Health and Caring Science, Western Norway University of Applied Science, Førde, Norway; 16 Department of Behavioral Health, Reliant Medical Group, Worcester, MA USA; 17 Department of Psychology, University of Copenhagen, Copenhagen, Denmark; 18 Institute of Psychology, University of Osnabrück, Salzburg, Austria; 19 Institute of Synergetics and Psychotherapy Research, Paracelsus Medical University, Salzburg, Austria

**Keywords:** Progress feedback, Routine outcome monitoring, Measurement-based care

## Abstract

We face increasing demand for greater access to effective routine mental health services, including telehealth. However, treatment outcomes in routine clinical practice are only about half the size of those reported in controlled trials. Progress feedback, defined as the ongoing monitoring of patients’ treatment response with standardized measures, is an evidence-based practice that continues to be under-utilized in routine care. The aim of the current review is to provide a summary of the current evidence base for the use of progress feedback, its mechanisms of action and considerations for successful implementation. We reviewed ten available meta-analyses, which report small to medium overall effect sizes. The results suggest that adding feedback to a wide range of psychological and psychiatric interventions (ranging from primary care to hospitalization and crisis care) tends to enhance the effectiveness of these interventions. The strongest evidence is for patients with common mental health problems compared to those with very severe disorders. Effect sizes for not-on-track cases, a subgroup of cases that are not progressing well, are found to be somewhat stronger, especially when clinical support tools are added to the feedback. Systematic reviews and recent studies suggest potential mechanisms of action for progress feedback include focusing the clinician’s attention, altering clinician expectations, providing new information, and enhancing patient-centered communication. Promising approaches to strengthen progress feedback interventions include advanced systems with signaling technology, clinical problem-solving tools, and a broader spectrum of outcome and progress measures. An overview of methodological and implementation challenges is provided, as well as suggestions for addressing these issues in future studies. We conclude that while feedback has modest effects, it is a small and affordable intervention that can potentially improve outcomes in psychological interventions. Further research into mechanisms of action and effective implementation strategies is needed.

## Introduction

We face a global crisis in routine mental health care. Across the world, markers of behavioral and emotional distress have risen sharply (Kuehn, 2020). This has resulted in the need for greater access to care, which can be at least partially facilitated with virtual means (Douglas et al., 2020; Shore et al., 2020). There is also a need for more effective care in everyday settings. While psychological interventions are generally effective in reducing symptoms and improving functioning in patients (Barkham & Lambert, 2021; Lambert, 2013), effectiveness varies substantially based on where care is delivered. In randomized clinical trials (RCTs), an average of 67% of patients are reliably improved by the end of treatment (Hansen et al., 2002). In a direct comparison between efficacy trials and routine practice data, results showed patient outcomes for the latter to be at an approximate 12% disadvantage compared with patient outcomes in trials (Barkham et al., 2008).

Progress feedback is considered to be one of the most promising methods to improve outcomes in routine care (Barkham et al., 2023a, b; Bickman, 2008; Kazdin, 2008; Wampold, 2015) and has been endorsed for use in routine care by professional organizations, insurance companies, and governments (Boswell et al., 2023). While the principle of providing progress feedback has increasingly been viewed as part of good practice, the reality remains that adoption and implementation by practitioners is low. The aim of the current review is to provide a summary of the existing evidence base for the use of progress feedback, including theorized mechanisms of action and implementation strategies.

### What is Progress Feedback?

*Progress feedback* refers to the ongoing monitoring of patients’ treatment response with standardized measures and routinely feeding this information back to the clinician and/or patient, who can subsequently decide whether alterations in the treatment are indicated. It has been implemented in a wide variety of mental healthcare settings worldwide, ranging from college counseling centers to community mental health care and crisis inpatient settings (De Jong et al., 2018; Lambert et al., 2001; Probst et al., 2013). Progress feedback has also been used with youth populations (Bergman et al., 2018; Bickman et al., 2011, 2016; Connors et al., 2023; Jensen-Doss et al., 2020; Van Sonsbeek et al., 2021) and been applied to problems like common mental health disorders, personality disorders, substance dependence, eating disorders, and psychotic disorders (Crits-Christoph et al., 2012; Davidsen et al., 2017; De Jong et al., 2018; Probst et al., 2013; Schiepek et al., 2016; van Oenen et al., 2016; Bovendeerd et al., 2022). The practice of progress feedback has been described using various labels, such as *measurement-based care* (Scott & Lewis, 2015), *patient-reported outcome measurement* (Kendrick et al., 2016), *routine outcome monitoring* (Wampold, 2015), and *outcome feedback* (Delgadillo et al., 2017). In this review we adopt *progress feedback* as the generic term to encompass all variants.

In more advanced progress feedback systems, a patient’s progress is compared to a trajectory derived using data from previous patients. This enables the calculation of *expected treatment response* curves that visually represent the boundaries within which typical symptom fluctuations are observed over time (Finch et al., 2001). A warning signal or alert is provided when a patient’s current symptoms are significantly more severe than expected, which is referred to as a case that is “not on track” (NOT; Harmon et al., 2007). NOT cases are at risk of poor treatment outcomes, such as enduring symptoms after treatment or deterioration (Lutz et al., 2006). Some systems supplement NOT signaling technologies with *clinical support tools* (CSTs; e.g., additional questionnaires, instructions, manuals, videos) designed to assist clinicians in identifying and addressing problems that may be interfering with treatment progress (Harmon et al., 2007; Lutz et al., 2019). Although most feedback systems are now premised on the use of session-by-session measures, the systems are heterogeneous in terms of the measures adopted, the algorithms used to identify NOT cases, and the decision-rules to deal with poor progress in therapy.

## Evidence Base

### Current Evidence Base

Since its introduction in 2001 (Lambert et al., 2001) more than sixty studies on the effectiveness of progress feedback in psychotherapy have been conducted and eleven meta-analyses of studies applying feedback in mental health care have been published, with divergent findings and conclusions (Bergman et al., 2018; De Jong et al., 2021; Kendrick et al., 2016; Knaup et al., 2009; Lambert et al., 2003, 2018; Østergård et al., 2020; Pejtersen et al., 2020; Rognstad et al., 2023; Shimokawa et al., 2010; Tam & Ronan, 2017; see Table 1). Table 1 shows that overall effect sizes of progress feedback found in meta-analyses range between negligible and medium effect sizes. Effect sizes tend to be stronger in NOT cases (*d* = 0.17 to *d* = 0.53; see Table 1), and when CSTs are added for NOT cases (*d* = 0.35 to 0.49; De Jong et al., 2021; Lambert et al., 2018).

The most comprehensive meta-analysis, summarizing the results of 58 studies, suggests that the overall effect of feedback on symptom reduction is small but robust (fail-safe *N* = 3695), both for the full samples (*d* = 0.15) and NOT cases (*d* = 0.17; De Jong et al., 2021). In addition, feedback significantly reduces the odds of treatment dropout (*OR* = 1.19). This meta-analysis also demonstrated that the effect of progress feedback is larger for studies conducted in the US, compared to those conducted in other countries, possibly because of differences in care systems and training programs (De Jong et al., 2021). Different types of feedback systems seem to work best for different type of populations, with simpler systems working better for milder populations and more advanced systems working better for more severe populations (De Jong et al., 2021; Østergård et al., 2020).

Most meta-analyses have been limited in their ability to test for moderators and mediators of the effectiveness of progress feedback, largely because few studies report the same variables, or do not report on potential moderators and mediators at all. Systematic reviews have aimed to fill that gap by synthesizing evidence from different studies, even if they do not measure exactly the same constructs (Carlier et al., 2012; Davidson et al., 2015; Gondek et al., 2016; Krägeloh et al., 2015). All these reviews concluded that progress feedback has great potential to improve clinical outcomes, but that its effects may depend on feedback and treatment setting characteristics. Krägeloh and colleagues (2015) created a typology of the complexity of different progress feedback systems, concluding that more advanced systems that apply *expected treatment response curves* and CSTs tend to produce better outcomes than simpler models. Davidson and colleagues (2015) state that feedback seems mainly effective in the treatment of mild to moderate psychological problems, or at least that in very severe populations the usefulness of progress feedback might be limited. This conclusion is supported by later studies that found no effects and sometimes even potentially detrimental effects of feedback in very severe populations, such as inpatient personality disorder treatment and severe mood disorders (De Jong et al., 2018; Errázuriz & Zilcha-Mano, 2018).

### Limitations of the Evidence Base

Akey problem in the feedback literature is that the methodological quality of studies is often relatively poor (Kendrick et al., 2016). To some extent, applying a less methodologically rigid research design is understandable given that feedback studies are often pragmatic trials that study the effects of progress feedback as implemented into routine practice. However, several improvements in study design are warranted. In particular, in the majority of studies, outcome assessments are not conducted independent of the therapy process, and the measure on which feedback is provided is often also the primary outcome measure (Østergård et al., 2020). This problem raises the possibility that the effects of feedback could be partly explained by social desirability bias or demand characteristics of the situation where a patient is primed to closely attend to a specific outcome measure repeatedly. Furthermore, many feedback trials are likely to be statistically underpowered, since the meta-analytic evidence indicates that we should expect small effect sizes, thus requiring larger sample sizes than those observed in most trials (Barkham, 2023; Barkham & Lambert, 2021). In addition, many trials suffer from fidelity issues. When clinicians do not utilize feedback as intended (De Jong et al., 2012), it may substantially reduce the effect size of the intervention compared to treatment as usual.

Moreover, in the majority of studies, there is limited control over the extent to which the feedback provided is actually utilized by the therapist and the consistency with which it is applied (Delgadillo et al., 2022). Feedback systems are frequently offered as packages with many different features, and we do not know much about which features are essential for feedback to be effective. Most studies assessed progress feedback and compared it to no feedback; only some studies have compared the effectiveness of different features of feedback systems (Harmon et al., 2007; Slade et al., 2008). Progress feedback intervention features may include aspects related to the measures (e.g., frequency, type), how interpreted data are visualized and provided to the clinician, the inclusion of comparative benchmarking, and decision support tools. Systems vary in ‘ease of use’ characteristics, such as administration methods for patient-reported measures (e.g., paper-and-pencil or digital, pre-determined and/or tailored), monitoring fidelity for implementation support, and aggregating data for management purposes.

Reporting of key implementation metrics (e.g., measure completion, feedback viewing) is needed to inform benchmarking for quality of implementation (Bickman et al., 2016; Douglas et al., 2023b; Lewis et al., 2019). Future studies should also focus on a broader spectrum of outcome and progress measures (Boswell, 2020). Research on progress feedback should consistently report on dropout and treatment duration, and consider alternative outcomes that may be more relevant to patients’ lived experience, such as quality of life and work/school functioning (Metz et al., 2019; Wolpert et al., 2017). There is also a need for more research on progress feedback in specialized populations, such as child and adolescent psychotherapy (Bergman et al., 2018; Connors et al., 2023; Douglas et al., 2023b; Jensen-Doss et al., 2020) and settings, such as telehealth (Van Tiem et al., 2022).

### Emerging Understanding of Potential Mechanisms of Action

Theoretical models of progress feedback suggest it may impact outcomes by focusing the attention of the clinician on discrepancies between their own case conceptualization and what is reported on measures (Riemer & Bickman, 2011; Sapyta et al., 2005). This could serve to change clinician expectations about patient progress and provide new information useful to treatment planning. It has also been suggested that patients’ understanding of their problems, engagement in treatment, and therapeutic alliance may be improved through assessment and feedback (Finn & Tonsager, 1997). Only a few studies have attempted to investigate the potential mechanisms of action of progress feedback. This section provides hypotheses as to how progress feedback might work.

### Feedback Focuses Attention of the Clinician

One hypothesis is that feedback might be effective because it draws the attention of the clinician to cases that are not progressing well and are at risk of poor outcomes, thereby enabling them to identify and resolve problems more quickly. Clinicians seem relatively poor at being able to recognize cases that are at risk of poor outcomes (Hannan et al., 2005; Hatfield et al., 2010) and may be unaware that some patients are not benefitting from therapy or are worsening. Recent research by Janse and colleagues (2023) supports the notion that frequent feedback may benefit clinicians with a high level of self-efficacy by correcting their biases towards overestimating their own effectiveness. A feedback system could help clinicians overcome this over-optimistic bias by providing a warning signal, which then draws the attention of the clinician and focuses their attention and efforts on supporting patients who are not progressing well. This hypothesis suggests that the “signal” is the active element that is associated with the effects of feedback, by focusing attention and priming problem-solving efforts.

One RCT investigated this hypothesis in a context where the only difference between intervention and control groups was that clinicians assigned to the feedback condition had access to risk signaling technology, which led to improved outcomes relative to the control group for NOT cases (Delgadillo et al., 2018). However, clinicians using the feedback also had been trained in how to resolve obstacles to clinical improvement in consultation with supervisors, so the “signal effect” hypothesis is only partially supported. Other recent studies have not found beneficial effects of feedback signaling alone and suggest that it can be demoralizing in the absence of specific instructions on how to resolve clinical problems (Errázuriz & Zilcha-Mano, 2018). Hence, it is plausible that supplementing signaling technology with clinical instructions and training is essential to maximize the effects of progress feedback.

### Feedback Changes Clinicians’ Expectations

Another potential mechanism is that clinicians’ expectations are altered through the feedback, given their overly optimistic outcome expectations. In a quintessential study, Hannan et al. (2005) asked clinicians to identity the patients in their caseload who would be likely to deteriorate. Clinicians identified 3 out of 550 patients (0.01%) as likely to deteriorate, much lower than the known base rate for their practice. Actual outcomes indicated that 40 patients (7%) had deteriorated by the end of therapy. Thus, clinicians grossly overestimated their patients’ outcomes.

De Jong and colleagues (2024) tracked clinicians’ outcome expectations over time as part of an RCT in which one control group and two feedback groups were compared. In each condition, clinicians rated the odds that their client would improve reliably by the end of therapy at session one, five, and ten. In the control condition, clinicians overestimated the odds of treatment success at each time point, compared to actual outcomes. In the first feedback group, in which clinicians received progress feedback without warning signals, clinicians had similar expectations as in the control group, but because outcomes had improved due to the feedback, their expectations were closer to actual outcomes. In the second feedback group, in which feedback with warning signals and CSTs were provided, outcomes improved, but expectations had decreased as well by session ten. Thus, feedback that uses warning signals and CSTs might work in part because clinicians’ overoptimistic outcome expectations are altered, perhaps making them more alert to intervene when patients do not progress well. However, more research is needed on the topic to draw more definite conclusions.

### Feedback Provides New Information

Feedback may also simply provide clinicians with new information that they would not otherwise have gathered. Douglas et al. (2015) found that when feedback is provided, topics that are rated by patients as important were discussed sooner in the course of therapy compared with when no feedback was provided. Moreover, studies applying CSTs that assess the therapy process (e.g., working alliance, motivation) have also found larger effects than obtained in studies using feedback systems not applying CSTs (Shimokawa et al., 2010). In general, there seems to be a tendency for feedback systems to yield larger effect sizes when they assess multiple aspects of the therapy process.

An interesting new study makes a strong case for the inclusion of treatment process measures in progress feedback (Camacho et al., 2021). At each daily session, patients completed measures of symptoms and wellbeing, and also answered questions about how they were applying skills (e.g., cognitive reappraisal) they were learning during a 10-day cognitive behavioral therapy (CBT) group treatment. There was a predictive relationship between self-reported application of CBT skills and patient outcomes. Greater confidence in and use of CBT skills learned in treatment were associated with greater wellbeing and lower symptoms. The inclusion of treatment process measures, such as application of therapy skills, in progress feedback would allow clinicians early in treatment to target where there is low use or lack of confidence in skills and thus bolster the likelihood of a positive treatment response. This is consistent with recommendations to use progress feedback to guide adaptation and tailor care (Georgiadis et al., 2020), especially in terms of reactive flexibility, where the clinician adjusts treatment as it progresses to be responsive to emerging factors.

### Feedback Enhances Patient-Clinician Communication

In a systematic review on feedback effects in somatic and mental health care, Carlier and colleagues (2012) found that progress feedback improved patient-provider communication both in the short and longer term. A qualitative study of patients and clinicians’ views of clinical feedback systems found that such systems supported collaboration, enabled open conversation, and enhanced interpersonal processes (Moltu et al., 2018). Patients generally find feedback informative (Delgadillo et al., 2017; Lutz et al., 2015) and qualitative studies of feedback utilization reported that completing the questionnaires helped patients focus on what they want to talk about in the session with the clinician, as well as helped them learn which coping skills are more or less effective in managing their symptoms and problems (Delgadillo et al., 2017; Unsworth et al., 2012).

The use of progress feedback to inform patient-clinician communication is complex, partially because it involves at least two actors and the interaction between them. Clinicians may vary in their skill at integrating feedback information with other ongoing processes in treatment and ability to make it meaningful for patients. Thus, the use of feedback in clinical communication is likely both a mechanism of action for how feedback works but also will influence (and be influenced by) the quality of implementation. A systematic review and meta-analysis of qualitative studies of both clinician and patient perspectives on feedback highlighted the use of feedback data to facilitate communication (Låver et al., 2023). The authors suggest that patient-reported data go beyond objective measures of functioning by supporting exploration, reflection, and shifts in care. Two qualitative studies focused on patient preferences related to valuing progress feedback in treatment (Holliday et al., 2021; Solstad et al., 2021). Both studies emphasized the need for clinicians to routinely share some information about progress feedback in a meaningful way, yet also to tailor how that information is shared to the needs and preferences of the patient. A list of eight recommendations for communicating with patients about progress feedback is summarized in Table 2. The use of progress feedback should be considered a clinical skill, which needs to be both taught and practiced to reach its full potential.

There is even greater complexity for patient-clinician communication when considering youth psychotherapy, which typically involves multiple actors in the treatment process. With youth and caregiver respondents, the clinician must exercise clinical skill and ethical practice around confidentiality in weighing how to utilize each distinct perspective in treatment (Connors et al., 2023; Douglas et al., 2023b; Jensen-Doss et al., 2020). It has been established that youths and caregivers provide uniquely valuable information about treatment targets and outcomes (De Los Reyes et al., 2023). Such discrepancies in feedback data may represent opportunities for the clinician to engage youths and their caregivers in conversations about their different perspectives. Case examples indicate that this type of clinician-facilitated communication using feedback data contributes to shared understanding, collaborative decision-making, and empowered voice for young people (Connors et al., 2023; Douglas et al., 2023b; Jensen-Doss et al., 2020). Objectively, there may also be differences in how respondent measures predict treatment outcomes. For example, two RCTs conducted by Bickman and colleagues found that the impact of feedback on youth mental health outcomes differed by reporter, with clinician-report of youth symptoms showing greater improvement in both studies (Bickman et al., 2011, 2016) and youth-reported symptoms improving faster in only the former study.

The potential for progress feedback to enhance communication is especially relevant in today’s world with the recent global pandemic and the rapid rise in telehealth services. Telehealth is here to stay, and the addition of progress feedback as a transtheoretical and transdiagnostic intervention may enhance systematic ongoing monitoring, treatment engagement, and therapeutic alliance in the context of the virtual encounter (Douglas et al., 2020). In addition to strategies summarized in Table 2, recommendations for using progress feedback in telehealth include technological accessibility issues (e.g., digital means for sending and completing measures, ability to show screens) and measurement considerations (e.g., individualized items, risk monitoring, and therapeutic alliance) (Douglas et al., 2020; Van Tiem et al., 2022).

### Feedback Enhances the Therapeutic Alliance

There is emerging evidence that the therapeutic alliance (TA) is influenced by progress feedback. Brattland and colleagues (2019) found that in the first two months of treatment, the TA increased more when progress feedback was used in treatment, compared to when no progress was provided. In addition, an increase in TA was associated with better treatment outcomes, suggesting that TA moderated the effect of progress feedback on treatment outcomes (Brattland et al., 2019). The influence of feedback to direct attention and communication between a clinician and patient (and caregivers) is likely to improve collaborative and patient-centered care (Carlier et al., 2012), and treatment engagement (e.g., fewer drop-outs from care, De Jong et al., 2021), which aligns with Bordin’s (1979, 1994) tripartite definition of TA as agreement on therapeutic goals, the tasks to achieve those goals, and the bond between the clinician and patient (and/or caregivers). There is also emerging evidence for the addition of measures of treatment process (e.g., TA) as part of a multidimensional approach to progress feedback (Connors et al., 2021; Jensen-Doss et al., 2020). Whether a standard part of the measurement package (e.g., Bickman et al., 2011, 2016) or queued up as part of additional clinical decision support based on symptom trajectory (e.g., De Jong et al., 2021), the inclusion of such measures can improve clinicians’ ability to recognize TA ruptures and shifts in engagement to directly address them in treatment (Chen et al., 2018).

### Implementation Issues and Strategies

Despite robust findings of progress feedback’s effects on treatment outcomes, the implementation of progress feedback in routine clinical practice is often low (Jensen-Doss et al., 2018; Patterson et al., 2006). Although routine outcome measurement is mandatory in many countries, measuring outcomes does not necessarily mean that this information is being used to inform treatment. Several studies have shown that even in RCTs, up to half the clinicians do not use the progress feedback to inform treatment decisions (De Jong et al., 2012; Simon et al., 2012). In their 2011 RCT, Bickman and colleagues found that one-third of clinicians never even viewed a progress feedback report. Based on a content analysis of clinicians’ written notes about using feedback in sessions, Casline and colleagues (2023) found they incorporated feedback data into about one-fifth of sessions. When data were used, they were used inconsistently and typically for short-term purposes (e.g., current symptoms) rather than long-term decision-making. Recent findings by Van Sonsbeek et al. (2023) suggest that implementing progress feedback in large mental health care organizations presents challenges related to clinician characteristics and a lack of adequate guidance, which leads to limited impact on client outcomes. Deisenhofer and colleagues (2024) provide a substantial review of the barriers to implementing the broad class of precision methods in personalizing the psychological therapies that includes feedback and the possible ways of resolving them.

Good integration into everyday clinical practice seems to be a crucial factor in the effectiveness of progress feedback on improving outcomes, which strongly increases with better implementation (Bickman et al., 2016; Brattland et al., 2018). McLeod and colleagues (2022) propose a framework for progress feedback approaches that focuses on meaningful use to improve care while also allowing for flexibility in how feedback is implemented based on characteristics and goals of the practice setting. Barber and Resnick (2022) introduced the *Collect, Share, Act* model as a valuable approach for integrating and utilizing progress feedback in clinical sessions. Douglas and colleagues (Douglas et al., 2023b; Jensen-Doss et al., 2020; Youn et al., 2023) expand on this approach to encompass the organizational setting and larger health care system. They propose a simple strategy of identifying the value that feedback can bring to promote use at each level, from use of one point-in-time in a session to aggregating data across sessions, patients, clinicians, and ultimately within and across services and organizations.

Overall, there is a small but growing literature on barriers and facilitators that influence the adoption and sustained use of progress feedback systems (De Jong & De Goede, 2015; Douglas et al., 2016, 2023a; Jensen-Doss et al., 2020; Lewis et al., 2019). Implementation is complex and requires attention at multiple levels including the individual patient, clinician, organization, and system level. Recent qualitative and quantitative studies of clinician and patient perspectives suggest four themes that impact implementation of feedback systems in mental health settings: practical considerations, attitudinal concerns, interventional issues (e.g., selection of measures), and context (e.g., organizational-level variables; Boyce et al., 2014; Douglas et al., 2023a; Lewis et al., 2019; Town et al., 2017).

Practical concerns relate to perceived difficulties with administering outcome measures and integrating the feedback into typical clinic workflow, such as the time burden, unclear roles or guidelines, technological difficulties, and the need for training and support. Barriers at individual patient- and clinician-levels are often related to practical concerns about infrastructure and leadership support, in addition to negative attitudes about the usefulness and acceptability of incorporating measures as part of routine practice (Gleacher et al., 2016; Rye et al., 2019; Walter et al., 1998). For example, therapists and patients have expressed concerns about undermining the therapeutic relationship, the measures being too simplistic to reflect the typical complexity of individual cases, and how data may be used to evaluate the therapist’s performance. There is also a need for more research to better understand how progress feedback systems can be better designed to fit diverse populations and settings. In addition to determining cultural equivalence in different patient populations, recent research suggests that clinicians’ cultural identity and perceptions of fit may influence access to progress feedback (Boswell et al., 2023). Shifts in how progress feedback is structured and implemented may improve feasibility and acceptance in diverse settings.

The selection of measures is particularly important. Some research indicates patients’ preferences for measures that assess positive aspects of treatment progress such as quality of life and wellbeing (Crawford et al., 2011; Kilbourne et al., 2010; Lyon et al., 2016). In several qualitative studies of clinicians’ attitudes toward feedback systems, the perceived relevance (or lack thereof) of the data to ongoing clinical care has been reported (Wolpert et al., 2016). For example, in a case example (Douglas et al., 2023a, b clinician reported that one of her youth patients found the symptom measure helpful but wished that there could also be questions relevant to an emerging gender dysphoria. Individualized measures may be useful to tailoring feedback for specific populations, such as goal attainment scales for older adults (Clair et al., 2022; Giovanetti et al., 2021) and goal-based outcome measures and problem rating scales for youth patients (Edbrooke-Childs et al., 2015; Weisz et al., 2011). More positive attitudes toward feedback systems were linked to more frequent use of feedback (De Jong et al., 2012; Jensen-Doss et al., 2018; Rye et al., 2019). These findings suggest that it is important to address concerns form clinicians and patients regarding the implementation of measures.

Organizational-level factors that should be considered when implementing progress feedback include adequate attention to resources for (continuous) training (Edbrooke-Childs et al., 2016), matching the feedback information flow to organizational infrastructure and priorities (Douglas et al., 2016; Jensen-Doss et al., 2020), and being mindful of potential misuse of feedback data as part of performance evaluation (De Jong, 2016). Feedback systems may also be useful in supporting existing organizational quality processes, such as clinical supervision (Fullerton et al., 2018) and quality improvement initiatives (Devine et al., 2013), where aggregated data can help identify areas of improvement for both clinicians and organizations. Progress feedback can help to identify cases for ongoing clinician reflection and learning as an important part of deliberate practice, a method aimed at improving the therapeutic skills of clinicians (Chow et al., 2015). Douglas and colleagues (2023a) suggest that assessing organizational management and clinical leadership perspectives is key to informing better implementation of progress feedback. A qualitative analysis of clinician, supervisor, and leader interviews from nine mental health organizations across three countries resulted in a framework for describing barriers and facilitators related to people, processes, structures, and contexts. These are both internal to organizations and external, representing health care system and national policy influences on implementation, such as national mandates or reimbursement strategies (e.g., Roe et al., 2021). While such a systemic approach to progress feedback implementation is quite complex, ultimately it is necessary to help us not only understand but also influence behavior change and organizational learning for better feedback practice.

## Conclusion

Having gained considerable momentum in the last decade, research has yielded compelling evidence of the potential for progress feedback to improve treatment outcomes in routine care for patients with common mental health problems, although it may be contra-indicated for patients with severe mental disorders or personality disorders. But it is not a panacea and the evidence base has limitations. However, potential mechanisms of action may include focusing the attention of the clinician, ‘rightsizing’ clinician expectations for improvement, providing new and relevant clinical information, and enhancing patient-centered communication. Promising approaches to strengthen progress feedback interventions include more advanced systems with signaling technology, clinical problem-solving tools, a broader spectrum of outcome and progress measures, together with the recognition that feedback-informed treatment is both a pan-theoretical psychotherapeutic method and a clinical skill that may improve with training, implementation support, and deliberate practice.

**Table 1:** Effect sizes reported by meta-analyses on the effectiveness of progress feedback on symptom reduction

					Full sample		NOT cases	
	Setting	Feedback system(s)	N	# of studies	*d* or *g*	95% CI	*d* or *g*	95% CI
Lambert et al., 2003	CCC	OQ	2,605	3	n.r.	n.r.	0.39	n.r.
Knaup et al., 2009	CCC, outpatients, day patients and inpatients	Various	4,540	12	n.r.	n.r.	n.r.	n.r.
Shimokawa et al., 2010^1^	CCC, outpatients	OQ	6,151	6	n.r.	n.r.	0.53	[0.28, 0.78]
Kendrick et al., 2016	CCC, outpatients	OQ and PCOMS	3,696	12	0.07	[-0.01, 0.16]	0.22	[0.09, 0.35]
Tam & Ronan, 2017	Youth, age 10–19	Various	3,804	10	0.28	[0.01, 0.55]	n.r.	n.r.
Bergman et al., 2018^2^	Youth, age 11–18	Various	858	5	n.r.	n.r.	n.r.	n.r.
Lambert et al., 2018	CCC and outpatient clinics	OQ and PCOMS						
- OQ System			7,509	10	0.14	[0.08, 0.20]	0.33	[0.25, 0.41]
- PCOMS			2,113	8	0.40	[0.29, 0.51]	n.r.	n.r.
Østergård et al., 2020	CCC and outpatient clinics	PCOMS	2,910	18	0.27	[0.14, 0.41]	0.21	[-0.11, 0.53]
Pejtersen et al., 2020	CCC, outpatients, and day patients	PCOMS	1,697	13	0.13	[0.001, 0.26]	n.r.	n.r.
De Jong et al., 2021	CCC, outpatient, day patients and inpatients	Various	21,699	58	0.15	[0.10, 0.20]	0.17	[0.11, 0.22]
Rognstad et al., 2023	Any primary care	Various	13,807	39	0.14	[0.08, 0.21]	0.29	[0.11, 0.46]

Note NOT = not on track; CCC = college counseling center; OQ = Outcome Questionnaire; PCOMS = Partners for Change Outcome Management System; n.r. = not reported; N/A = not applicable. ^1^ efficacy analyses; ^2^ reports on relative risk of percentage of improved cases, dropout, and number of missed sessions

**Table 2:** Recommendations for communicating with patients about progress feedback

♣ At a minimum, provide a clear and ongoing rationale for progress feedback to make it meaningful to the patient’s treatment experience
♣ Address feedback at each session but tailor to the patient’s needs and preferences
♣ Consider giving only brief updates on progress, especially when results are similar across time, then move on to clinical content
♣ Connect progress (or lack thereof) on scores to patient skills and strategies covered in treatment
♣ Consider using visual representations, such as graphs, to share feedback
♣ Explore and negotiate how and when to address progress feedback to allow fit with patient preferences
♣ Some patients may benefit from direct and frequent discussion of results, which can be used to structure sessions or initiate conversation about difficult topics
♣ Other patients may prefer less frequent, or more subtle indirect communication (i.e., guiding the conversation toward important topics but without explicitly referring to items or scores)
